# Supplementary material for: How do world and European standard populations impact burden of disease studies? A case study of disability-adjusted life years (DALYs) in Scotland
Source: Arch Public Health. 2020 Jan 3;78:1. doi: 10.1186/s13690-019-0383-8 (PMC6941317; doi:10.1186/s13690-019-0383-8)
Supplement: Supplementary file 2 — Additional file 2. Summary of GBD 2017 results from GBD country profile for the United Kingdom. [file 13690_2019_383_MOESM2_ESM.docx]

**Figure S1. Top 10 causes of DALYs in 2017 and percent change, 2007-2017, Scotland, all ages, number**


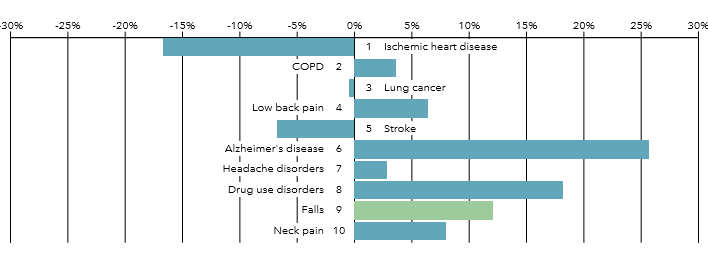


Causes of disease/injury ranked based on descending order of number of Disability-Adjusted Life Years (DALYs); Source: GBD 2017 country profile for Scotland (Accessed 27 September 2019)

**Figure S2. Top 10 causes of DALYs in 2017, Scotland, all ages, age-standardised rate (GBD WSP), by location**


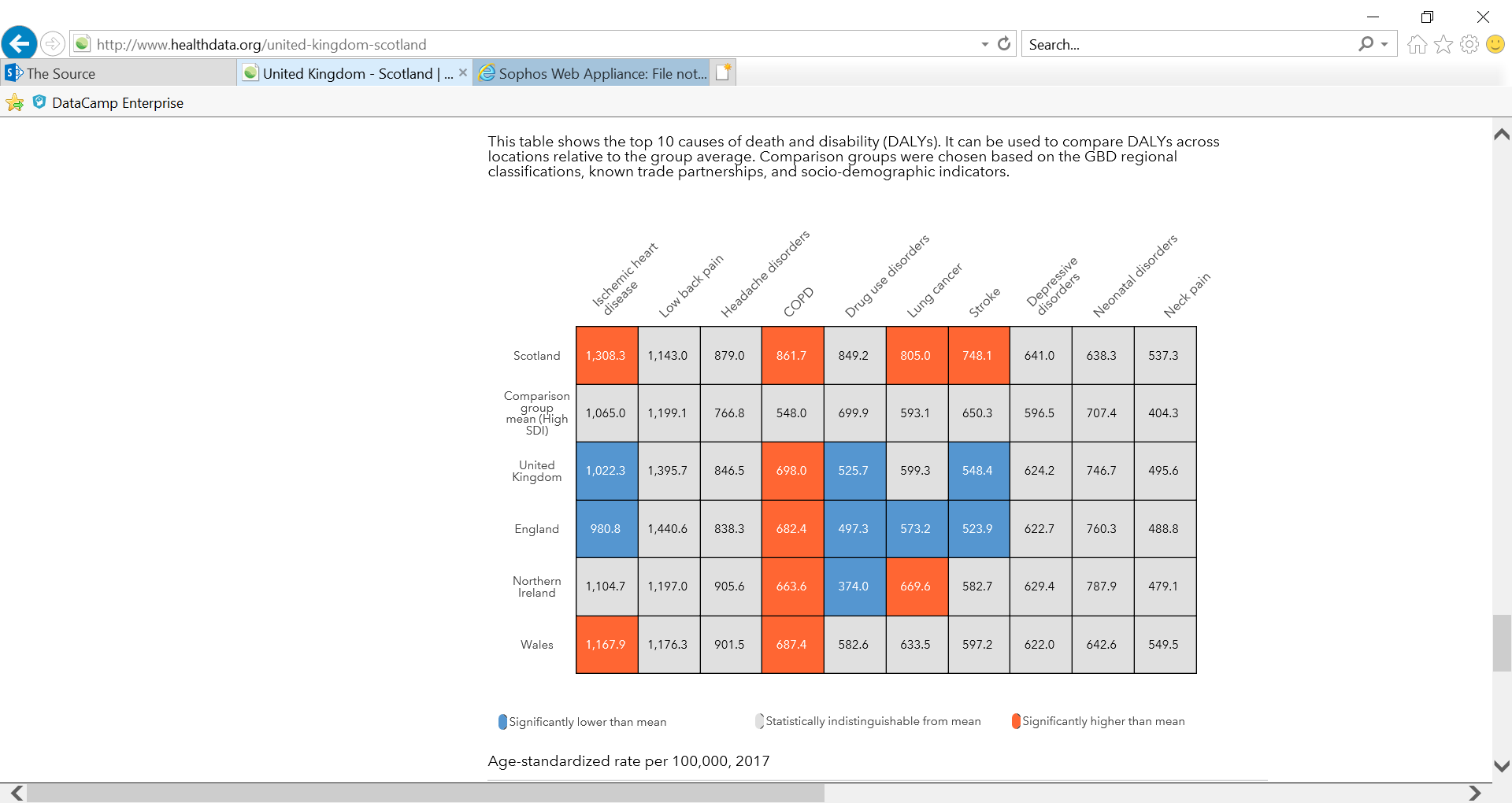


Comparisons are made relative to the group mean; Comparison groups were chosen based on the GBD regional classifications, known trade partnerships, and socio-demographic indicators; Source: GBD 2017 country profile for Scotland (Accessed 27 September 2019)
